# Treatment of Chronic Traumatic Diaphragmatic Hernia Based on Laparoscopic Repair: Experiences From 23 Cases

**DOI:** 10.3389/fsurg.2021.706824

**Published:** 2021-07-15

**Authors:** Qiaonan Liu, Li Luan, Guangyong Zhang, Bo Li

**Affiliations:** ^1^Department of Hernia and Abdominal Wall Surgery, Shandong Provincial Qianfoshan Hospital, Shandong University, Jinan, China; ^2^Department of Gastrointestinal Surgery, Shandong Provincial Qianfoshan Hospital, Shandong University, Jinan, China

**Keywords:** laparoscopy, diaphragmatic hernia, laparotomy, thoracotomy, muscle flap

## Abstract

**Background:** We aimed to investigate the safety and effectiveness of laparoscopic repair for treating chronic traumatic diaphragmatic hernia (CTDH).

**Methods:** In this retrospective analysis, we included 23 cases with CTDH underwent laparoscopy in our hospital between June 2015 and October 2019 was performed. The patient characteristics were recorded. We compared the diameter of hernia ring, surgery duration, intraoperative bleeding volume, means of repairing, as well as the follow-up data.

**Results:** All the patients underwent laparoscopic diaphragmatic hernia repair, without conversion to laparotomy or thoracotomy. The operation time ranged from 60 min to 200 min (mean, 108.04 ± 42.93 min). The blood loss volume ranged from 10 to 300 ml (mean volume, 63.48 ± 71.69 ml). The postoperative hospital stayed ranged from 5 to 15 days (mean, 6.22 ± 2.11 days). The patients were followed up for 1–50 months (mean, 17.5 ± 10.90 months). No recurrence of diaphragmatic hernia was found.

**Conclusions:** Laparoscopic repair of CTDH is featured by fast recovery, high security, and effectiveness. Reducing the hernia contents and close of the hernia ring are crucial for the surgery that is performed based on the size and location of the diaphragmatic hernia.

## Introduction

Traumatic diaphragmatic hernia (TDH), a spectrum of disease processes based on temporal patterns from acute to chronic (1), is a rare disease encountered in 0.8–6% of blunt trauma and more than 17% of thoraco-abdominal-penetrating trauma (2). Grimes divided TDH into 3 phases based on a similar schema designed by Carter and colleagues: 1. Acute 2. Latent 3. Obstructive (3).

As the symptoms of TDH are usually insidious, over 30% of the patients with diaphragm injures would not present symptoms immediately after trauma. Abdominal organs herniated into the chest cavity would lead to clinical symptoms of different systems including chest symptoms (e.g., chest pain, tightness, shortness of breath, and dyspnea), abdominal symptoms (e.g., abdominal pain, nausea, vomiting, stop flatus and defecation, and other manifestations of intestinal obstruction, acid reflux, and belching), as well as the symptoms (e.g., chronic anemia). In cases of strangulation and necrosis in herniate organs, there might be signs of peritonitis, which is a great threat to the life of patients (4, 5).

Diaphragmatic hernia patients with no spontaneous remission are suggested to undergo surgery upon diagnosis (4, 6, 7). To date, its treatment is highly relied on transthoracic, transabdominal and thoracoabdominal approaches (4, 5, 8, 9). However, there are disputes on the efficiency of these treatment options. For the cases with a large hernia ring, there might be challenges in the tissue repair even after the coverage using multiple meshes, leading to a high possibility of recurrence. To our best knowledge, rare studies focused on the feasibility of autologous muscle flaps for the closing of the hernia ring even of a large size forming bridge grafting. In this study, we retrospectively analyzed 23 cases of CTDH and summarized the surgical skills and experiences based on laparoscopic repair.

## Materials and Methods

### Patients

Twenty-three patients with CTDH underwent laparoscopic herniorrhaphy in our department from June 2015 to October 2019 were included in this study. These confirmed with diaphragmatic hernia induced by trauma were eligible to this study upon confirmation using the X ray, CT scan, upper gastroenterography and MRI. The exclusion criteria were as follows: (a) those with severe immune and/or endocrine disorders, hepatic or renal insufficiency, with poor tolerance to surgery; (b) those with severe mental disorders, or those with severe cognitive dysfunction; (c) those with severe coagulation disorders. This was a retrospective analysis, and the informed consent was waived by the ethics committee of our hospital.

Most of the patients diagnosed with chronic TDH had been admitted to the hospital at the time of the trauma and had been discharged without a diagnosis of TDH after examination. A small part of cases presented mild diaphragmatic injuries, and they decided to choose conservative therapy.

### Pre-surgical Protocol

Before surgery, each patient underwent determination of cardiopulmonary function and coagulation test to exclude the contraindications for surgery and anesthesia. Gastrointestinal decompression was performed to the cases with intestinal obstruction. In addition, fasting fluid infusion and other symptomatic treatment were given before surgery. To improve the cardiopulmonary function, antibiotic was administrated to the patients with pulmonary infection.

### Surgical Procedures

Each patient was in a fasting state before surgery, followed by insertion of gastric tube. General anesthesia was conducted using the double-lumen endotracheal intubation with a reverse Trendelenburg modified lithotomy position.

**Figure 1:**
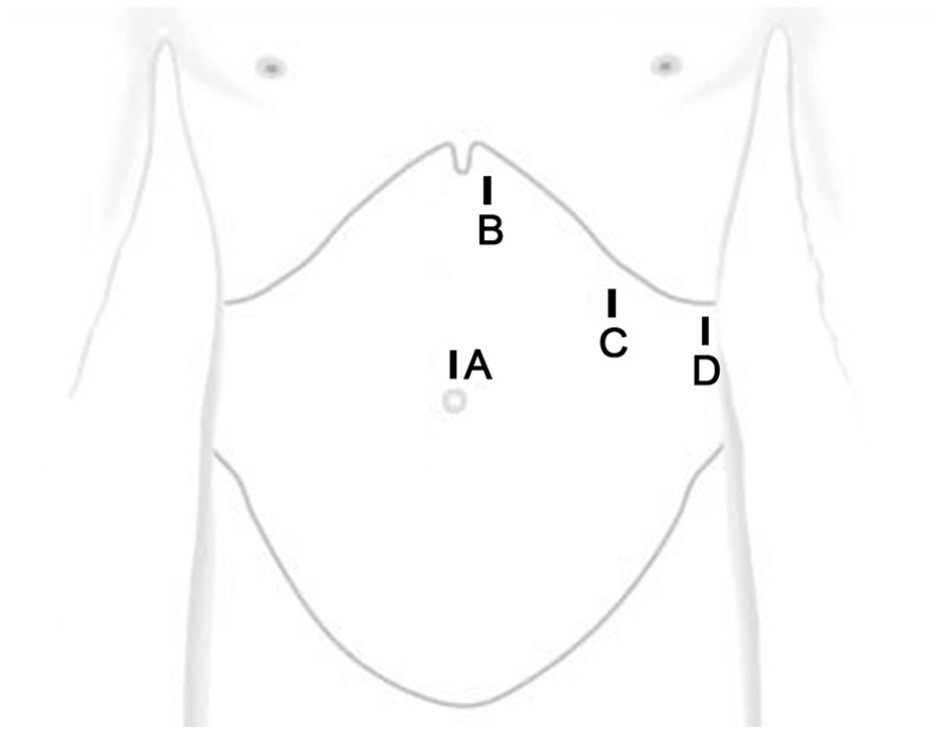
Port placement sites.

**Figure 2:**
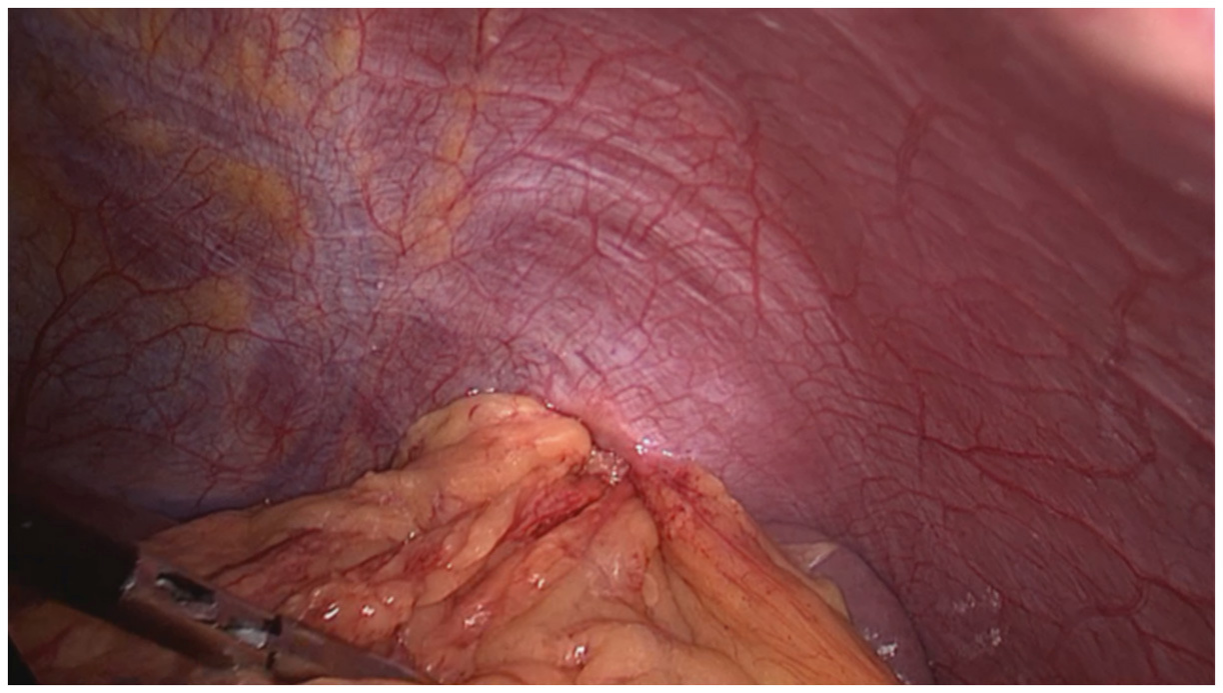
Reducing hernia contents.

A longitudinal incision (10 mm) was made above the umbilicus, followed by insertion of laparoscope. Afterwards, trocars with a dimension of 5 mm, 12 mm and 5 mm were placed at the position that was about 3 cm to the left side beneath xiphoid process, 3 cm beneath costal margin of left midclavicular line, and 3 cm beneath the costal margin of left anterior axillary line, respectively (Figure 1). The size and location of the diaphragmatic hernia were confirmed, together with organ herniation into the thoracic cavity and adhesion to the surrounding tissues. The adhesion was carefully isolated along the hernia ring, and the omental tissues herniated into the thoracic cavity were dissected. Afterwards, the exudate in the hernia sac was aspirated. If necessary, 1–2 short gastric vessels can be cut. The organ herniated into the thoracic cavity was returned to the abdominal cavity with an atraumatic grasping forceps (Figure 2). In addition, attention should be paid to avoid grasping the organ during reduction. In cases of any difficulties, the hernia ring can be incised in a radiated pattern (1–2 cm) away from the pericardium with an electric coagulation hook to achieve release. The hernia ring was repaired by continuous suturing with non-absorbable sutures. For the lesions with a diameter of larger than 5 cm, a suitable anti-adhesion mesh was utilized to reinforce the defect area. Fixation of the mesh was performed using surgical tacks, tissue glue and suture. For the treatment of 3 cases with a hernia ring of > 10 cm, there was obvious fibrosis, it is still difficult to conduct direct suturing. Therefore, a pedicle muscle flap was obtained from the anterior abdominal wall near the hernia ring. Then the flap was turned around to cover the defect, and then 2–0 prolene suture was used to fix the pedicle muscle flap and the contralateral hernia ring. Finally, mesh was utilized to repair diaphragmatic hernia and abdominal wall defect. A drainage tube was inserted into the residual lumen of the hernia sac.

The gastric tube was removed on postoperative day 2. The abdominal drainage tube was removed when the drainage volume was <30 ml.

### Follow-Up

The patient was followed up for 1–50 months (mean, 17.5 ± 10.90 months). Each patient was required to receive physical examination at month 1, 3, 6, 12, and 24 after surgery. During the follow-up, each patient received CT and upper gastrointestinal radiography to monitor the recurrence and complications.

## Result

### Patient Characteristics

In this study, 23 cases were finally included. The demographic data, operation time, mechanism of injury, preoperative complications, the size of defect, type of repair, and follow-up time were listed in Table 1. None of the cases showed necrosis of the hernia contents.

**Table 1:** Patient characteristics.

**Patient No**.	**Age, y**	**Gender**	**Causes**	**Preoperative complication**	**Size, cm**	**Type of repair**	**Secondary injury**	**Blood loss, ml**	**Operating time, min**	**Post-operative complications**	**Follow-up time, month**
1	51	Male	Vehicle collision	Chest pain	7	Suture + Mesh	Small intestine	80	170	None	50
2	46	Male	Vehicle collision	Dyspnea, chest pain	6.5	Suture + Mesh	None	30	120	None	12
3	37	Male	Vehicle collision	Dyspnea, anemia	8	Suture + Mesh	None	35	110	None	24
4	60	Female	Fall	Anemia, lung infection	13	Autologous tissue + mesh	Splenic hemorrhage, colon injury	100	200	Pleural effusion	36
5	27	Male	Vehicle collision	Chest pain, low fever	4	Suture	None	10	80	None	24
6	31	female	Vehicle collision	Dyspnea	5	Suture + Mesh	None	20	70	None	18
7	41	Male	Vehicle collision	Low fever, anemia	7	Suture + Mesh	Small intestine	45	90	Chest pain	5
8	63	Male	Vehicle collision	Gastrointestinal obstruction	7.5	Suture + Mesh	Small intestine	90	130	Pleural effusion	24.5
9	50	Female	Vehicle collision	Lung infection, anemia	8	Suture + Mesh	None	65	100	Low fever	12
10	70	Female	Fall	Gastrointestinal obstruction, low fever	7.5	Suture + Mesh	Hemorrhage of greater omentum	75	130	None	24
11	72	Male	Vehicle collision	Dyspnea, gastrointestinal obstruction	14	Autologous tissue + mesh	Small intestine rupture, splenic hemorrhage	240	200	Chest pain, pleural effusion	24
12	21	Male	Fall	None	3	Suture	None	15	70	None	12
13	39	Male	Vehicle collision	None	5	Suture + Mesh	None	20	80	None	18
14	38	Female	Fall	Low fever, anemia	6	Suture + Mesh	Stomach	30	100	None	24
15	68	Male	Vehicle collision	Lung infection	6	Suture + Mesh	None	10	60	Chest pain	18
16	51	Male	Others	Stomach upset	4	Suture	None	20	80	None	12
17	36	Female	Fall	None	3	Suture	Stomach	60	90	None	12
18	63	Female	Vehicle collision	Anemia, lung infection	6	Suture + Mesh	Small intestine rupture	100	140	Pleural effusion	12
19	19	Male	Vehicle collision	None	3	Suture	None	20	60	None	18
20	45	Male	Vehicle collision	Anemia, lung infection	5	Suture + Mesh	None	40	65	Lung infection	5
21	71			Gastrointestinal obstruction	8	Suture + Mesh	Obstructive necrosis of transverse colon	300	160	Lung infection, pleural effusion	1
22	67	Male	Vehicle collision	Stomach upset, cough	11	Autologous tissue + mesh	None	25	120	None	12
23	31	Male	Vehicle collision	Lung infection, anemia	5	Suture + Mesh	Stomach	30	60	None	5

### Treatment Efficiency

**Figure 3:**
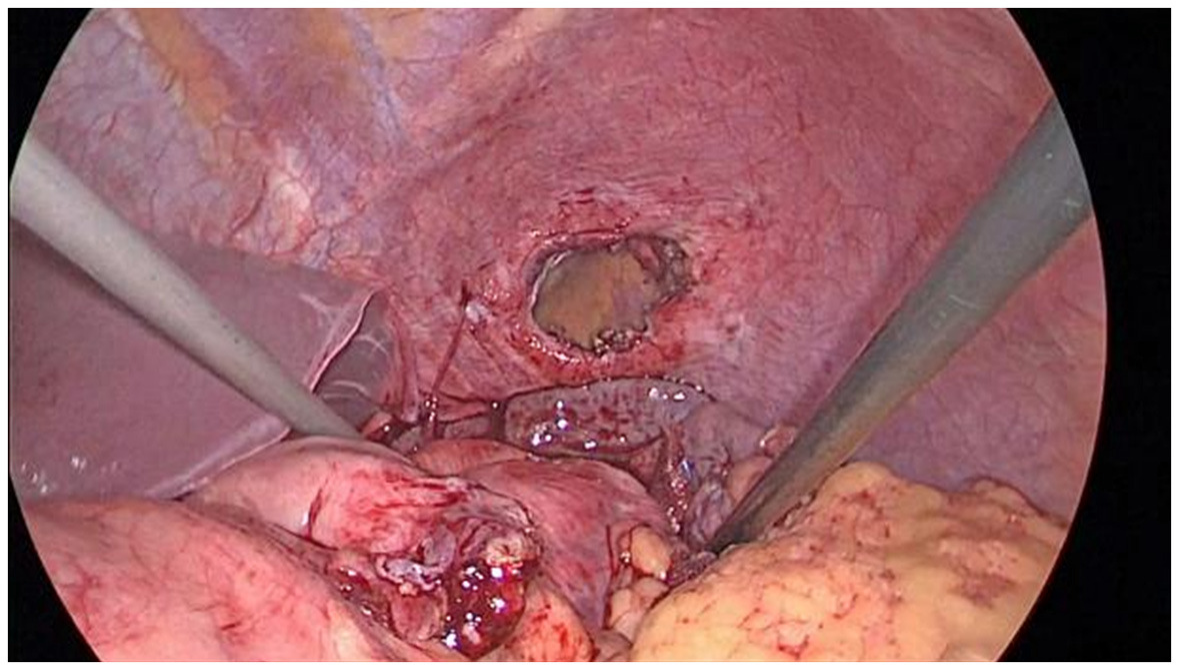
The hernia ring and surrounding area should be exposed at least 5 cm after reducing the contents of the hernia.

Twenty-three patients in this group underwent diaphragmatic hernia repair via laparoscopic technique, with no necessity to convert to laparotomy or thoracotomy. Table 1 showed the operation time, blood loss, follow-up time, and post-operative complications of the 23 cases. In this study, the herniated tissues were recovered successfully in 20 cases. Three cases showed severe adhesion to the hernia sac with the herniated stomach, colon and spleen tissues, which was returned after release of adhesions. Four cases showed seromuscular damages in the gastric wall. One case showed seromuscular rupture in small intestine and colon, which were sewed up under laparoscope. One case presented splenic capsule rupture and bleeding, and then hemostasis was given by electric coagulation hook after reduction. Upon reduction of the hernia contents, the hernia ring and peripheral area (5 cm) were completely exposed (Figure 3). Subsequently, the diaphragmatic defect was intermittently sutured with 2–0 non-absorbable sutures. No one showed recurrence of diaphragmatic hernia. Patients with postoperative complications showed attenuation after conservative treatment.

**Figure 4:**
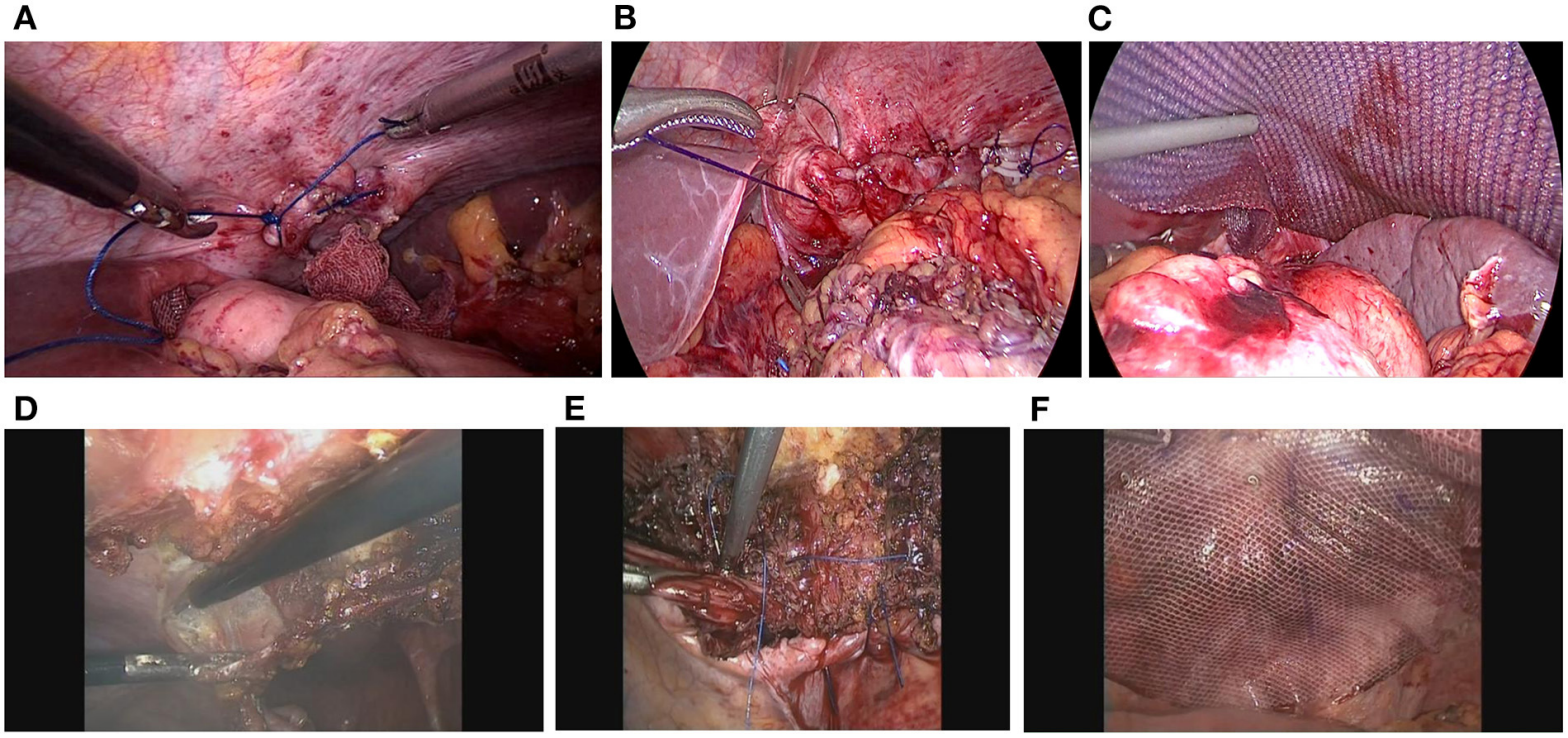
Suture of the hernia ring with various sizes. **(A)** For the hernia ring with a diameter of <5 cm, single suture was given. **(B,C)** Suturing and mesh fixation were utilized for the treatment of hernia ring in a range of 5–10 cm. **(D–F)** The muscle flap formed by the incision of anterior abdominal wall tissues, together with suture and mesh, were utilized to close the giant hernia ring with a diameter of ≥10 cm.

The hernia ring with a diameter of <5 cm can be directly sutured and repaired (Figure 4A). In the presence of a hernia ring with a diameter of ≥5 cm, anti-adhesion mesh with an appropriate size was used for fixation at a position that was about 3–5 cm beyond the edge of hernia ring. In this group, Sepramesh (Bard) was used for repair, and fixation was conducted using the Fixation Device Covidien [ProTack((SP))TM((/SP))] (Figures 4B,C). During the fixation, glue and continuous suturing were utilized in the position that was close to the heart, pericardium, aorta and esophagus (10). Three cases showed huge hernia rings (≥10 cm) which could not be sutured. Pedicle muscle flaps with appropriate sizes were formed by separating the anterior abdominal wall tissues adjacent to the hernia rings, and then the flaps were inverted to close the contralateral hernia ring (Figures 4D–F). The diaphragmatic hernia and abdominal wall defect (i.e., the muscle flap free site) were repaired simultaneously using an anti-adhesive mesh, avoiding the “bridging repair” of the diaphragm. An abdominal drainage tube was placed in the residual cavity of the hernia sac, and no closed thoracic drainage tube was placed.

### Follow-Up

For the follow-up, all the patients were followed up for 17.5 ± 10.90 months (1–50 months). For the disease recurrence and complications, CT and upper gastrointestinal radiography indicated that no one showed recurrence and severe complications.

## Discussion

About 90% diaphragmatic hernia occur on the left side, while the right diaphragm is usually protected by the liver with a small chance of TDH (5, 11–13). Our data were in line with these literatures. All the patients with CTDH may not present spontaneous remission (14). Besides, the area of diaphragmatic injury will be extended over time (15). On this basis, these patients are recommended to receive surgery as early as possible upon diagnosis.

Compared with congenital diaphragmatic hernia (e.g., hiatal hernia), severe adhesion of herniate organs was noticed in CTDH, which resulted in vague landmark in the planes and normal anatomical structures. Therefore, it is essential to properly dissect the hernia sac to avoid injury to the surrounding viscera (16).

As diaphragmatic defect edges were weak, thin and fragile (17), there was usually a large tension when suturing the diaphragm, especially the part adjacent to the central of the hiatus. Thus, mesh repair was required for these cases (18). According to our experiences, for the hernia ring with a diameter of <5 cm, mesh may not be necessary if the diaphragmatic defect could be easily sutured. Nevertheless, mesh is recommended for the management of hernia ring with a diameter of ≥5 cm. After taking the special anatomy of diaphragm into consideration, the diaphragmatic defect should be closed to avoid “bridging repair.” In this study, we innovatively utilized the muscle flap formed by the incision of anterior abdominal wall tissues to close the giant hernia ring (10 cm). In addition, the diaphragmatic hernia and abdominal wall defect near the muscle flap free site were repaired with a big anti-adhesion mesh. These procedures were easy to perform and showed reliable efficiency for the repair.

Compared with laparotomy and thoracotomy, laparoscopic repair is superior in treating CTDH as it involves minimally invasive procedures, including less trauma, faster recovery, and shorter hospital stay (5). Compared with thoracoscopic surgery, laparoscopy contributed to the complete exploration of the entire abdominal cavity, which could illustrate the presence of adverse events such as hemorrhage and gastrointestinal rupture (19). For the cases with hemorrhage and gastrointestinal rupture, the lesions could be managed directly under the laparoscopic guidance, which was superior to the thoracoscopy. Simultaneously, it is much more difficult to fix the mesh to the thoracic surface of the diaphragm than to the abdominal surface of the diaphragm. The transabdominal approach is more exact for the repair effect. Indeed, the disease course of diaphragmatic hernia is comparatively longer, together with obvious adhesion, which triggered the difficulty in organ recovery into the abdominal cavity. Therefore, there is a harsh technical demanding for the treatment of CTDH under the guidance of laparoscopy. In cases of severe adhesion and difficulty in return of hernial content, gastrointestinal tract injury or bleeding during the surgery, the patients are recommended to undergo immediate laparotomy or thoracotomy.

Indeed, there are some limitations in our study. The sample size is not large, and the follow-up duration is not long. Besides, the interval between the initial injury and TDH symptoms is comparatively long, and then we could not obtain the clinical data for the first presentation. In future, more studies involving a large sample size and a long follow-up are required to further illustrate the efficiency of such regimen for the treatment of CTDH.

## Conclusion

Laparoscopic technique is effective for treating CTDH as it triggers generation of clear surgical fields. Meanwhile, it is able to manage the adverse events simultaneously. The key point for the surgery is the reduction of hernia contents and the closure of hernia ring. In this study, the pedicle muscle flap we utilized was formed by freeing the anterior abdominal wall tissues close to the hernia ring and suturing the defect with the contralateral hernia ring tissue, which avoided the presence of “bridging repair” caused by incomplete hernia ring closure. Such technique is worthy of being widely popularized in the clinical application.

## Data Availability Statement

The original contributions presented in the study are included in the article/supplementary material, further inquiries can be directed to the corresponding author/s.

## Ethics Statement

The studies involving human participants were reviewed and approved by the Ethics Committee of Qianfoshan Hospital of Shandong University. The patients/participants provided their written informed consent to participate in this study.

## Author Contributions

QL wrote the manuscript. BL revised the manuscript. GZ did the data analysis. LL did the data collection. All authors read and approved the final manuscript.

## Conflict of Interest

The authors declare that the research was conducted in the absence of any commercial or financial relationships that could be construed as a potential conflict of interest.
